# Regulation of Alloantibody Responses

**DOI:** 10.3389/fcell.2021.706171

**Published:** 2021-07-08

**Authors:** Anita S. Chong, Peter T. Sage, Maria-Luisa Alegre

**Affiliations:** ^1^Section of Transplantation, Department of Surgery, University of Chicago, Chicago, IL, United States; ^2^Renal Division, Transplantation Research Center, Brigham and Women’s Hospital, Harvard Medical School, Boston, MA, United States; ^3^Section of Rheumatology, Department of Medicine, University of Chicago, Chicago, IL, United States

**Keywords:** Tfh, Tfr, alloantibodies, Bregs, transplantation, tolerant B cells, transplant tolerance

## Abstract

The control of alloimmunity is essential to the success of organ transplantation. Upon alloantigen encounter, naïve alloreactive T cells not only differentiate into effector cells that can reject the graft, but also into T follicular helper (Tfh) cells that promote the differentiation of alloreactive B cells that produce donor-specific antibodies (DSA). B cells can exacerbate the rejection process through antibody effector functions and/or B cell antigen-presenting functions. These responses can be limited by immune suppressive mechanisms mediated by T regulatory (Treg) cells, T follicular regulatory (Tfr) cells, B regulatory (Breg) cells and a newly described tolerance-induced B (TIB) cell population that has the ability to suppress *de novo* B cells in an antigen-specific manner. Transplantation tolerance following costimulation blockade has revealed mechanisms of tolerance that control alloreactive T cells through intrinsic and extrinsic mechanisms, but also inhibit alloreactive B cells. Thus, the control of both arms of adaptive immunity might result in more robust tolerance, one that may withstand more severe inflammatory challenges. Here, we review new findings on the control of B cells and alloantibody production in the context of transplant rejection and tolerance.

## Introduction

Transplantation tolerance remains an important goal to reduce side effects associated with lifelong immunosuppressive drugs and to diminish the incidence of chronic rejection that might arise from suboptimal immunosuppression. Both T cell-mediated and antibody-mediated mechanisms have been invoked in acute and chronic rejection, though some controversy exists as to whether the appearance of donor-specific antibodies (DSA) is simply a marker of incompletely suppressed T effector and T follicular helper (Tfh) cell responses, or if DSA independently cause rejection (Chong et al., 2019). Several animal models of transplantation tolerance have been developed, and proof of principle data exist that select patients can develop transplantation tolerance (Brouard et al., 2012; Morris et al., 2015; Miller et al., 2017; Savage et al., 2018). New genetic and flow cytometric tools have increased our ability to probe the mechanisms that underscore tolerance when it is successfully induced, though most of the work has focused on how alloreactive T cells are controlled. Mechanisms of T cell transplantation tolerance appear to parallel endogenous immunological mechanisms ensuring T cell self-tolerance, including clonal deletion, anergy, cell-extrinsic suppression and aborted B cell help. Whereas lack of B cell responses might result from insufficient T cell help, new studies are revealing an active tolerance crosstalk between T and B cells. Indeed, T follicular regulatory (Tfr) cells can dominantly suppress B cell responses, Bregs can inhibit T cell responses and promote Treg development, and a novel subset of tolerance-induced B (TIB) cell can infectiously inhibit the responses of naïve B cells in an antigen-specific manner. This active tolerization of the alloreactive B cell compartment may be desirable to achieve a more robust transplantation tolerance than that obtained when only the T cell compartment is regulated. This review will focus on new findings involving Tfr cells, Breg cells and alloreactive TIB cells and their roles in the control of alloimmunity and transplant rejection.

## T Follicular Regulatory (Tfr) Cells

### Tfr Cells in Antibody Regulation

Antibody responses are tightly controlled by the immune system. Tfh cells promote antibody responses by providing costimulatory and cytokine signals to promote B cell effector responses (Crotty, 2019). Humans with mutations in Tfh effector genes as well as mice with genetic deletion of the same genes have severely impaired antibody responses (Crotty, 2019). Tfr cells are a subpopulation of FoxP3^+^ T regulatory cells that express the chemokine receptor CXCR5 and can gain access to the B cell follicle to regulate B cell responses (Fonseca et al., 2019; Sage and Sharpe, 2020; Wing et al., 2020). Although CXCR5 was originally thought to be absolutely essential for the migration of Tfr cells to the B cell follicle, newer data suggest other signals may also be involved (Vanderleyden et al., 2020). Tfr cells have a somewhat overlapping transcriptional program as Tfh and Treg cells, and follow similar differentiation cues as Tfh cells (Hou et al., 2019). The shared transcriptional program between Tfr cells and Tfh/Treg cells has hindered in-depth functional studies.

Although Tfr cells have been studied for over 10 years, the precise functions of these cells have been somewhat controversial. Human correlation studies suggest inverse correlations between Tfr cells (as well as Tfr:Tfh ratio) and antibody responses in settings of vaccination, infection and autoimmunity (Sage and Sharpe, 2016; Deng et al., 2019). In some limited settings, such as Sjogren’s syndrome, the Tfr/Tfh ratio may positively correlate with ectopic lymphoid structure formation, suggesting Tfr cells may also act as biomarkers for progressive disease stages (Fonseca et al., 2018). Uncovering the precise roles for Tfr cells in murine models has also been challenging. Early studies utilized adoptive transfer techniques and *in vitro* suppression assays to demonstrate that Tfr cells inhibit antigen specific antibody responses (Wollenberg et al., 2011; Sage et al., 2013, 2016). However, these strategies have technical limitations and lack physiological complexity. Recently, two *in vivo* models have been developed to study Tfr cells: Conditional deletion of Bcl6 in Treg cells (Treg^ΔBcl6^) and the Tfr-DTR mouse. The premise behind the Treg^ΔBcl6^ model is that the transcription factor Bcl6 may be necessary for most Tfr cell development and eliminating Bcl6 in all Treg populations would restrain Tfr cell differentiation from Treg cells. Although the specificity and potency of Tfr depletion in this model is unclear, a number of studies have demonstrated substantial increases in autoantibodies in Treg^ΔBcl6^ mice (Wu et al., 2016; Botta et al., 2017; Fu et al., 2018; Gonzalez-Figueroa et al., 2021). In contrast, Treg^ΔBcl6^ mice had minor (if any) increases in foreign-antigen specific antibody responses. Moreover, in some cases such as influenza infection, Treg^ΔBcl6^ mice have less influenza-specific antibody, suggesting Tfr cells may actually promote antibody responses in some settings (Lu et al., 2021).

As an alternative approach, we recently developed a Tfr-DTR mouse model in which Tfr cells can be deleted in an inducible manner. Using this mouse, we found that Tfr cells potently suppress germinal center formation to control both autoreactive and vaccine-specific antibody responses (Clement et al., 2019). We also found that Tfr cells potently control antigen-specific, as well as total, IgE responses. Increases in total IgE have subsequently been validated using Treg^ΔBcl6^ models (Gonzalez-Figueroa et al., 2021). Therefore, Tfr cells seem to have potent roles in controlling autoantibody responses, and the role of Tfr cells in restraining foreign antigen-specific antibody responses may depend on experimental context.

Tfr cells utilize a number of mechanisms to control B cell responses. The coinhibitory receptor CTLA-4 seems to be a potent mediator of Tfr suppression, and both downregulation of B7–1/B7–2 on B cells, as well as prevention of Tfh-B cell interactions, have been proposed as potential mechanisms (Sage et al., 2014; Wing et al., 2014). Although Tfr cells can produce the inhibitory cytokine IL-10, studies suggest that IL-10 production by Tfr cells may actually promote, not inhibit, B cell responses in some settings (Laidlaw et al., 2017). Mechanisms of Tfr suppression that are not shared with other regulatory cell subsets are less clear. However, a new study has uncovered neuritin as a possible Tfr-specific inhibitory molecule that may restrain B cell responses, although neuritin may suppress IgE/allergic responses more potently than IgG responses (Gonzalez-Figueroa et al., 2021).

### Tfr Cells in Transplantation Tolerance

The roles of follicular T cells in controlling solid organ transplant rejection are only beginning to be studied (Mohammed and Sage, 2020). In kidney transplant patients, the frequency of CXCR3^–^ Tfh cells (including Tfh2 and Tfh17 subsets) correlated with donor-specific antibody (DSA) responses and development of antibody-mediated rejection (AMR) (Chen et al., 2017). Similarly, in cardiac transplant patients, CXCR3^–^ Tfh cells were elevated 1 year after transplantation (Wang et al., 2020). In murine cardiac transplant models, Tfh cell expansion occurred prior to DSA responses suggesting Tfh cells may be an inducer (and biomarker) of rejection (La Muraglia et al., 2019). These studies suggest that Tfh cells may be driving AMR. Moreover, the ratio of Tfr to Tfh cells was attenuated in both kidney and cardiac transplant patients when compared to controls, and a decrease in the Tfr to Tfh ratio was associated with rejection in some transplant studies (Chen et al., 2017; Yan et al., 2019; Niu et al., 2020; Wang et al., 2020). These correlation studies suggest that Tfr cells may restrain Tfh and B cell responses to limit generation of pathogenic antibodies and prevent graft rejection, with the caveat that the ratios were quantified among circulating cells whereas regulation takes place in secondary lymphoid organs.

Elucidating the precise roles of Tfh and Tfr cells in controlling AMR after transplantation has been elusive due to a lack of tools. We recently developed a pre-clinical allogeneic kidney transplant model to assess the roles of Tfh and Tfr cells in controlling DSA and AMR. Using a Tfh-DTR model in which Tfh cells were deleted during transplantation we found that Tfh cells were essential for IgG DSA as well as AMR after kidney transplantation (Mohammed et al., 2021). We also performed allogeneic kidney transplantation in Tfr-DTR mice to assess the roles of Tfr cells in controlling AMR. Although we found that Tfr cells can limit IgG DSA using splenocyte alloantigen challenge models, we did not find substantial differences in severity of AMR during kidney transplantation when Tfr cells were deleted (Mohammed et al., 2021). However, one caveat to these experiments is that a full MHC mismatch kidney transplant model was used in which rejection rapidly occurs within 3 weeks. Therefore, it is unclear if it would be possible to detect quicker rejection by eliminating Tfr suppression. Studying the Tfr-DTR mouse in kidney transplant tolerance models will be important to determine if Tfr cells have important roles in promoting B cell tolerance during transplantation. *In vitro*, Tfr suppression of B cells results in epigenetic changes in B cells, diminishing their ability to produce class-switched antibody even after Tfr cells are no longer present (Sage et al., 2016). A somewhat similar phenotype may occur with alloreactive TIB cells acquiring a stable tolerance state during cardiac transplantation tolerance induced with costimulation blockade, although acquisition of tolerance was observed in Treg^ΔBcl6^ recipients (Khiew et al., 2020). That Tfr cells may be able to induce a tolerant-like state in B cells is supported by a recent study where tolerance induced by specific donor-recipient MHC combinations during kidney transplantation was mediated by FoxP3^+^ Treg cell subset(s) (Yang et al., 2020). Although the precise roles for Tfr cells in modulating transplantation tolerance are still being investigated, it is likely that Tfr cells have functional overlap with both Treg and Breg cells. Since Tfr cells differentiate mostly from Treg cells, it is possible that Treg promotion of B cell tolerance may be through a Tfr-dependent mechanism (Aloulou et al., 2016; Maceiras et al., 2017). Moreover, studies suggest that Breg cells may control Tfh cells, such that Tfr cells may synergize with Breg cells to promote tolerance by restraining Tfh-help to B cells (Lin et al., 2019).

## Regulatory B Cells

### Multiple Breg Subsets in Immunity

There is a substantial literature supporting a role for B cells in immune regulation, and numerous regulatory B cell subsets have been implicated (reviewed in Stolp et al., 2014; Alhabbab et al., 2019; Cherukuri et al., 2021a). For humans, these include the CD24^*hi*^CD38^*hi*^ immature transitional B cells (TBs) (Blair et al., 2010), CD24^*hi*^CD27^+^ B10-like B cells (Dilillo et al., 2010), CD27^+^CD38^*hi*^ plasmablast Bregs (Matsumoto et al., 2014), CD25^*hi*^CD71^*hi*^CD73^*lo*^ Br1 cells (Van De Veen et al., 2013), CD39^+^CD73^+^ Bregs (Saze et al., 2013), CD38^+^CD1d^+^IgM^+^CD147^+^ Bregs (Lindner et al., 2013), TIM-1^+^ Bregs (Aravena et al., 2017), and induced Bregs (Nouel et al., 2015). Likewise multiple Breg subsets have been reported in mice, including transitional T2 (Mauri et al., 2003), marginal zone (MZ) (Lenert et al., 2005), CD5^+^CD1d^+^ B10 (Kalampokis et al., 2013), TIM-1^+^ or CD9^+^ (Ding et al., 2011; Sun et al., 2015) B cells, as well as LAG-3^+^CD138^+^ plasmablasts/plasma cells (Shen et al., 2014; Fillatreau, 2018; Lino et al., 2018). The numerous Breg subsets together with the absence of Breg-specific lineage markers have prevented a unifying framework for understanding the origin of these cells.

At present, it appears that Bregs may exist as two distinct subsets; natural Bregs that are similar to thymus-derived natural Tregs and driven by a yet undefined lineage-determining transcription factor instructing their development in the bone marrow; and induced Bregs generated in the periphery from distinct B cell subsets upon encounter with antigen. In support of the former subset, Fillatreau (Fillatreau, 2018, 2019) reported on a novel subset of non-dividing regulatory LAG-3^+^CD138^+^ plasma cells that develop at steady state, even in germ-free mice, and that require B cell receptor (BCR) signals and Bruton tyrosine kinase (Btk) for their development. Importantly, these cells produce IL-10 within hours after stimulation and are therefore classified as “natural regulatory plasma cells.” In contrast, other subsets of Bregs, such as the B10 Bregs require exposure to immunizing antigen, and engagement of BCR, CD40 and/or TLR signaling (Zhang, 2013). An example are Bregs described by Rothstein and colleagues, where a 20–25-fold expansion of TIM-1^+^ Bregs across multiple canonical B cell subsets was observed in mice receiving an islet transplant and treated with a low-affinity anti-TIM-1 antibody (Yeung et al., 2015).

### Immunomodulatory Cytokines Produced by Bregs

Adding to the complexity of Breg immunobiology, each Breg subset appears capable of suppressing immune responses by shared, as well as distinct, mechanisms of action. The most widely implicated mechanism for Breg-mediated suppression is through production of the anti-inflammatory and immunomodulatory cytokine IL-10. IL-10 produced by B cells may inhibit the differentiation, or induce the apoptosis, of Th1 and Th17 cells while promoting Treg expansion, and alter DC maturation (reviewed in Stolp et al., 2014). In light of the absence of a Breg lineage marker, the expression of IL-10 has been widely used for defining Bregs. In a recent review, Cherukuri et al. (2021a) tabulated IL-10-producing cells within each mouse B cell subset upon a 5 h stimulation with LPS, PMA and ionomycin (LPIM). Only 1.7% of follicular B cells were IL-10-producing, but because they are the major B subset, they represented up to 44% of all B cells producing IL-10. A sizable 23% of MZ B cells were IL-10 producing, and represented 33% of all IL-10-producing B cells. In contrast, plasma cells by far had the highest percentage (74%) of IL-10-producing cells, but only represented 5% of all IL-10-producing B cells. Thus under *in vitro* LPIM stimulation, follicular and MZ B cells represented 77% of all IL-10-producing cells, although it remains to be determined if follicular and MZ B cells have similar dominant contribution *in vivo*.

Transitional B cells represent 4–10% of CD19^+^ B cells in healthy adults, and 15–20% of blood B cells in mice. Transitional B cells can be further subdivided into T1, T2 and T3 subsets, and can increase in percentages upon infection and autoimmunity (Giltiay et al., 2019). There is a sizable body of literature correlating the frequency of IL-10-producing transitional (T2-MZ) B cells with transplantation tolerance (Newell et al., 2010; Pallier et al., 2010; Sagoo et al., 2010; Chesneau et al., 2014), leading to the hypothesis that Bregs were being induced and might modulate T cell responses to mediate allograft tolerance. However, a critical examination of the B cell tolerance signature when compared with cells from patients on immunosuppression and controls was subsequently conducted by Rebollo-Mesa et al. (2016). The authors found that the percentage of transitional B cells increased significantly upon steroid withdrawal. Subsequently, azathioprine as well as calcineurin inhibitors were reported to inhibit the levels of transitional IL-10-producing B cells. These findings raised concerns that transitional B cells may not predict nor mediate tolerance and that their elevated levels in tolerant recipients might be a response to the absence of immunosuppression (reviewed in Alhabbab et al., 2019). Nevertheless, the possibility remains that these IL-10-producing transitional B cells might contribute to graft survival as a consequence of their anti-inflammatory properties (reviewed in Alhabbab et al., 2019).

In addition to IL-10, IL-35-producing B cells and plasma cells have been reported to be critical regulators of immunity during autoimmune and infectious disease settings (Shen et al., 2014; Wang et al., 2014). IL-35 is a member of the IL-12 family, and is comprised of Ebi3, which is shared with IL-27, and a p35 subunit, which is shared with IL-12. As a result, the quantification of IL-35-producing B cells is challenging, and attribution of a role for IL-35 using single-chain (Ebi3)-deficient mice is complicated by concomitant loss of IL-27, a cytokine that also has anti-inflammatory and regulatory properties. Similarly, elimination of p35 not only results in loss of IL-35 but also IL-12 (Kourko et al., 2019; Mirlekar et al., 2021). Furthermore, IL-35 is produced by CD4^+^FoxP3^+^Tregs, CD8^+^ Tregs, activated dendritic cells, and it signals through four receptors, IL-12Rβ2-IL-27Rα, IL-12Rβ2-IL-12Rβ2, IL-12Rβ2-GP130, and GP130-GP130 (Zhang et al., 2019). Thus, the complexity of IL-35 immunobiology requires that multiple controls be included to allow for a definitive demonstration that IL-35 is critical to the function of Bregs.

In the settings of allergy, cancer and autoimmune diseases, the regulatory function of B cells has been reported to be mediated by the potent immunoregulatory cytokine, TGFß (reviewed in Huai et al., 2021). In a model of anti-CD45RB-induced allograft tolerance, Zhao et al. (2010) reported that TGFß produced by B cells was necessary for tolerance, while IL-10 was not, and in fact appeared to promote chronic cardiac vasculopathy. Studies using a tolerance protocol of anti-CD45B and anti-TIM-1 to promote tolerance to islet allografts revealed an expansion of TIM-1^+^LAP^+^ B cells (Latency-associated peptide, LAP, is non-covalently associated with TGFß, and this complex is either secreted or deposited on the extracellular matrix), and TGFß was necessary for tolerance induction (Lee et al., 2012). Furthermore, TGFß, in combination with indoleamine 2,3-dioxygenase production by B cells, has been reported to regulate T cell proliferation, and promote the induction of TGFß and IL-10-producing Tregs *in vitro* (Nouel et al., 2015). Whether these TGFß-producing regulatory B cells represent a distinct phenotype and developmental lineage from the IL-10-producing Bregs requires further investigation.

Mirroring the observation that CD4^+^ T cells have different effector and regulatory phenotypes (Hollbacher et al., 2020), B cells can also differentiate into different effector B cell subsets (Beff) producing different pro-inflammatory cytokines capable of enhancing T cell responses independently of antibody production (Lund, 2008; Shen and Fillatreau, 2015). These observations collectively raise the possibility that the ratio of Bregs to Beff may serve as a rheostat for effector T cell responses. In support of this notion, Cherukuri et al. (2014) reported that B cells co-producing TNFα and IL-10 and having a low IL-10/TNFα ratio lacked regulatory function, whereas those having a high IL-10/TNFα ratio effectively suppressed T cell production of IFNγ and TNFα. They further reported that the more immature T1 B cell subset had a significantly higher IL-10/TNFα ratio than the more mature T2 subset, and that a higher IL-10/TNFα ratio in the T1 subset best predicted an absence of T cell-mediated rejection and a better renal graft outcome (Cherukuri et al., 2017, 2021b). Despite its strong predictive value, it is nevertheless unclear whether the balance of IL10 and TNFα in the T1 B cell subset is a biomarker of tolerance or causally inhibits T cell-mediated rejection. T1 B cells are short-lived immature B cells that undergo apoptosis upon BCR engagement, and only a fraction of T1 B cells enter the B cell follicle to access appropriate survival signals and mature into naïve B cells (Chung et al., 2003; Zhou et al., 2020). Thus, where in the secondary lymphoid organs transitional B cells might gain access to T cells to suppress their activation, remains to be more fully explored. Recently, Giltiay et al. (2019) suggested a new perspective on these newly formed B cells, proposing that they function as a first line defense by producing “natural” antibodies and as antigen-presenting cells, based on their distinct BCR repertoire, high expression of activation induced cytidine deaminase (AID), high sensitivity to pathogen-associated molecular patterns (PAMPS) and their ability to produce cytokines. Thus, it is possible that B cells with a transitional phenotype may rapidly differentiate into antigen-presenting cells with the ability to regulate T cell responses through cytokine production.

### Immunoregulation of T Cell Responses by Bregs Through Cognate Interactions

In addition to the production of immunomodulatory cytokines, there is accumulating evidence that cell surface molecules, such as PD-L1 and CD80/86, on Bregs, may engage coinhibitory molecules PD-1 and CTLA-4, respectively, to modulate T cell function (Blair et al., 2010; Siewe et al., 2013). Recently, Hasan et al. (2021) reported that T cell immunoreceptor with Ig and ITIM domains (TIGIT) is expressed on human memory CD19^+^CD24^+^CD27^+^CD39^+^IgD^–^IgM^+^CD1c^+^ B cells that are capable of mediating immune regulation *in vitro*. Although these B cells also expressed PD-L1, CD39/CD73 and TIM-1, as well as IL-10 and TGFß1, antagonism of TIGIT or granzyme B reduced their ability to suppress the production of IFNγ and IL-17 by anti-CD3/CD28-stimulated CD4^+^ T cells. These observations suggest that the engagement of inhibitory receptors by ligands expressed on Breg cells may result in the suppression of CD4^+^ T cell responses. Additionally, Breg cells were also able to inhibit monocyte-derived dendritic cell activation (CCR7, CD40, CD80/86, CD83, and ICOS-L) and their production of proinflammatory cytokines (IL-12A and IL-6) in response to LPS, thus highlighting a potential indirect mechanism of Breg-mediated control of T cell responses. Consistent with this possibility, Mohib et al. (2020) utilized 2-photon intravital microscopy to demonstrate cognate T:Breg cell interaction at the T:B border of spleens from immunized mice receiving antigen-pulsed Breg cells, and showed that a consequence of this T:Breg interaction is the reduced ability of the “regulated” T cells to interact with DCs. Whether Breg cells exert their regulation mainly within secondary lymphoid organs or are also able to migrate to sites of inflammation to modulate T cell responses, as has been reported for Tregs (Panduro et al., 2016; Campbell and Rudensky, 2020), remains to be clarified.

### Immunoregulation of Donor-Specific B Cell Responses by TIB Cells

**FIGURE 1:**
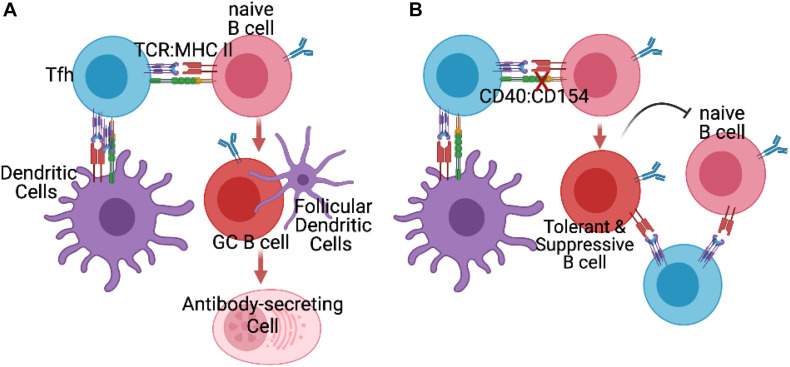
**(A)** In untreated recipients of allografts, Tfh cells generated by initial encounter with recipient dendritic cells presenting alloantigen engage to provide help to naïve alloreactive B cells, and facilitate their differentiation into germinal center (GC) B cells and antibody-secreting plasmablasts and plasma cells. **(B)** In the presence of blocking anti-CD154 (X), naïve alloreactive B cells fail to receive signals from CD40 and thus differentiate into tolerance-induced B (TIB) cells incapable of differentiating into GC B cells or antibody-secreting cells. Instead these B cells acquire the ability to prevent naïve donor-specific B cells from differentiating into germinal center or antibody-secreting cells, by as yet to be defined mechanisms.

In models of transplantation tolerance, much of the focus has been on the ability of Bregs to control T cells responses, and indirectly, control T-dependent B cell responses. As a result, other than testing for donor-specific antibody, investigations into the fate of donor-specific B cells have been neglected. We have recently addressed this gap in knowledge by examining the fate of donor-MHC-specific B cells in a mouse model of cardiac allograft tolerance induced by anti-CD154 and donor spleen cells. We showed that donor-specific B cells become dysfunctional and are intrinsically unable to differentiate into germinal center B cells and plasma cells, even upon transfer into non-tolerant mice and exposed to donor-antigen (Khiew et al., 2020; Figure 1). The transferred TIB cells were able to sense antigen and respond by increasing expression of CD69 and Glut-1, and augmenting mitochondrial mass and cell size, but exhibited lower proliferation rates and expression of AID, consistent with a cell-intrinsic block after metabolic reprogramming but before entry into germinal centers.

Remarkably, when TIB cells were co-transferred with naïve B cells, they were able to inhibit the naïve B cell responses in a donor-specific manner, though it is not known whether this inhibition involves direct cognate B:B cell interaction or occurs through close by-stander effects as a result of cognate interaction with a donor-specific T cell (Figure 1). These observations raise the novel possibility that B cells, upon encounter with alloantigen while being deprived of CD40 signals, become reprogrammed away from memory and plasma cell differentiation into B cells capable of suppressing other naïve B cells; this suppression is associated with thwarted differentiation of naïve B cells into germinal center B cells. It is notable that these TIB cells retain their ability to stimulate naïve antigen-specific T cell expansion and differentiation into Tfh cells, underscoring that such tolerant B cells were not mediating their effects though the overt suppression of T cell responses, as described for Bregs. The mechanism of donor-specific suppression of B cells responses by this unique subset of TIB cells requires further delineation.

## Conclusion

In addition to antibody secretion, B cells can play many roles in alloimmunity, including antigen presentation to T cells, and suppression of immune responses by Breg cells or donor-specific B cells differentiating into suppressor cells when encountering alloantigens in the absence of CD40 costimulation. New results suggest that DSA production is not only regulated by the balance between Tfh and Tfr cells, but also by TIB cells that can inhibit *de novo* activation of naïve alloreactive B cells in a donor-specific manner. Moreover, Bregs may also control alloreactive T cell responses, thus potentially reinforcing transplantation tolerance. How Bregs may affect the function and ratio of Tfh and Tfr cells, and how Tfh/Tfr may impact Bregs in alloimmunity, as is being suggested in autoimmunity (reviewed in Ding et al., 2021), remains to be investigated. A better understanding of these interplays in animal models during primary alloimmune responses but also during memory alloimmune responses, as well as investigations into which of these mechanisms are conserved in humans, may help design immunosuppressive regimens that protect the transplants long term, and perhaps facilitate life-long transplantation tolerance.

## Author Contributions

AC, PS, and M-LA conceived and wrote the review. All authors contributed to the article and approved the submitted version.

## Conflict of Interest

The authors declare that the research was conducted in the absence of any commercial or financial relationships that could be construed as a potential conflict of interest.
